# A forged ‘chimera’ including the second specimen of the protostegid sea turtle *Santanachelys* *gaffneyi* and shell parts of the pleurodire *Araripemys* from the Lower Cretaceous Santana Group of Brazil

**DOI:** 10.1186/s13358-023-00271-9

**Published:** 2023-05-05

**Authors:** Torsten M. Scheyer, Gustavo R. Oliveira, Pedro S. R. Romano, Dylan Bastiaans, Lisa Falco, Gabriel S. Ferreira, Márton Rabi

**Affiliations:** 1 Department of Palaeontology, University of Zurich, Karl Schmid-Strasse 4, 8006 Zurich, Switzerland; 2 Departamento de Biologia, Área de Ecologia, Laboratório de Paleontologia & Sistematica, Universidade Federal Rural de Pernambuco, Rua Dom Manuel de Medeiros S/No., Dois Irmãos, Recife, Pernambuco 52171-900 Brazil; 3 Departamento de Biologia Animal, Universidade Federal de Viçosa, Viçosa, Minas Gerais Brazil; 4 SCANCO Medical AG, Fabrikweg 2, 8306 Brüttisellen, Switzerland; 5 Senckenberg Centre for Human Evolution and Palaeoenvironment (HEP), Sigwartstrasse 10, 72076 Tübingen, Germany; 6 Department of Geosciences, University of Tübingen, Hölderlinstrasse 12, 72074 Tübingen, Germany; 7 Central Natural Science Collections, Martin Luther University Halle-Wittenberg, Domstraße 4, 06108 Halle (Saale), Germany

**Keywords:** Early Cretaceous, Santana group, Romualdo Formation, Marine turtle, *Protostegidae*, *Araripemydidae*

## Abstract

Fossils of Cretaceous sea turtles adapted to an open marine lifestyle remain rare finds to date. Furthermore, the relationships between extant sea turtles, chelonioids, and other Mesozoic marine turtles are still contested, with one key species being *Santanachelys* *gaffneyi* Hirayama, 1998, long considered the earliest true sea turtle. The species is an Early Cretaceous member of *Protostegidae*, a controversial clade either placed within or closely related to *Chelonioidea* or, alternatively, along the stem lineage of hidden-neck turtles (*Cryptodira*) and representing an independent open marine radiation. *Santanachelys* *gaffneyi* is one of the most completely preserved early protostegids and is therefore critical for establishing the global phylogenetic position of the group. However, the single known specimen of this taxon is yet to be described in detail. Here we describe a second specimen of *Santanachelys* *gaffneyi* from its type horizon, the Romualdo Formation (late Aptian) of the Santana Group of the Araripe basin, NE Brazil. The skeletal elements preserved include the posterior part of the skull, neck vertebrae, shoulder girdle, anterior-most and left/central part of the carapace with few peripherals, and plastron lacking most of the hyoplastra. The remaining part of the carapace was apparently completed by fossil dealers using an anterior part of the pleurodiran *Araripemydidae*, tentatively identified as a shell portion of cf. *Araripemys* *barretoi,* a more common Santana fossil turtle, among other indeterminate turtle shell fragments. The purpose of this paper is to report the repatriation of the specimen to Brazil and to provide a preliminary description.

**Supplementary Information:**

The online version contains supplementary material available at 10.1186/s13358-023-00271-9.

## Introduction

The Romualdo Formation and the underlying Ipubi and Crato formations of the Lower Cretaceous Santana Group, have so far yielded five turtle species in addition to two indeterminate pelomedusoid taxa (Oliveira et al., 2011; Romano et al., 2013). Of the five described species, all but *Araripemys* *barretoi* Price, 1973, which is known from more than 30 post-hatching specimens from the Romualdo and Crato formations (Carvalho et al., 2019; Limaverde et al., 2020; Meylan, 1996; Meylan and Gaffney, 1991; Oliveira and Kellner, 2005, 2017; Price, 1973; Romano et al., 2013), are considered to be rather rare faunal components so far only reported from the Romualdo Formation: the bothremydid pleurodiran *Cearachelys* *placidoi* is represented by seven specimens (Carvalho et al., 2019; Gaffney, 2001; Gaffney et al., 2006; Oliveira, 2007; Sena et al., 2021) and the euraxemydid pleurodiran *Euraxemys* *essweini* Gaffney, Tong and Meylan, 2006 by two specimens (Gaffney et al., 2006; Oliveira and Kellner, 2007; Romano et al., 2013), whereas the podocnemidoid pleurodiran *Brasilemys* *josai* Lapparent de Broin, 2000 and the protostegid pan-cryptodiran *Santanachelys* *gaffneyi* Hirayama, 1998 are represented so far only by their holotype specimens (Gaffney et al., 2006, 2011; Hirayama, 1998; Lapparent de Broin, 2000; Oliveira and Kellner, 2007; Oliveira and Romano, 2007).

Given its age and complete preservation, *Santanachelys* *gaffneyi* is a key taxon for understanding the evolution of adaptations to open marine lifestyle in sea turtles, but whether it is closely related to extant sea turtles (*Chelonioidea*) or represents a convergent marine radiation remains unclear. When first described, *Santanachelys* *gaffneyi* was found as the earliest diverging member of the extinct *Protostegidae* and formed a clade with leatherback sea turtles, *Dermochelyidae* (Hirayama, 1998). Kear and Lee (2006) reproduced these findings while sampling additional species, whereas Danilov and Parham (2006) and Joyce (2007) recovered *Santanachelys* *gaffneyi* with affinities to Mesozoic nearshore marine stem-cryptodirans such as *Solnhofia* *parsonsi* or other *Thalassochelydia* (see Anquetin et al., 2017 for a review of that clade), a signal that was also picked up in some subsequent large-scale analyses (e.g., Anquetin, 2012; Anquetin et al., 2015). Others placed *Santanachelys* in an even more stem-ward position, outside crown turtles (e.g., Sterli, 2010; Sterli and de La Fuente, 2011), but such a placement was not corroborated by more recent analyses. With the description of *Desmatochelys* *padillai* Cadena and Parham, 2015 from the Lower Cretaceous (upper Barremian–lower Aptian) of Colombia, *Santanachelys* *gaffneyi* has been no longer considered the stratigraphically oldest known marine turtle. In that study, the traditional placement of *Santanachelys* sensu Hirayama (1998) was recovered, but at the same time, low statistical support at the base of *Pan-Chelonioidea* and conflicting signals of spatiotemporal patterns and anatomy were used to question this topology (Cadena and Parham, 2015). Raselli (2018) recovered a monophyletic *Protostegidae* (with *Santanachelys* as sister to all remaining sampled protostegids) as sister group to extant *Cheloniidae* and *Dermochelys* (+ *Mesodermochelys*). The analysis of Evers and Benson (2019) again recovered protostegids in their traditional placement, but *Santanachelys* was found more deeply nested within the group. The analyses of Rabi and Kear (2016), Raselli (2018), Evers et al. (2019) and Gentry et al. (2019) corroborated the nested position within protostegids in a clade including *Notochelone* from Australia and *Rhinochelys* from Europe and, again, found *Protostegidae* closely related to crown-sea turtles, but representing their stem lineage. *Santanachelys* *gaffneyi* is hypothesised to be fully adapted to marine (open water) environment (Gentry et al., 2019), but its relation to crown-group sea turtles is yet to be rigorously established (Cadena and Parham, 2015).

Our understanding of the osteology of *S.* *gaffneyi* is based on the preliminary description of the holotype and single known specimen (Hirayama, 1998). Here we report a second specimen from the type horizon, a partial skeleton including an incomplete shell, posterior part of the skull, neck, and shoulder girdle elements. This fossil will contribute important data to a separate project on the detailed description of *S.* *gaffneyi* using high-resolution CT data. The goal of the present study is to provide a preliminary description, with focus on the shell elements, of the new specimen and document its repatriation to Brazil.

## Geological setting

The Romualdo Formation, formerly known as the Romualdo Member of the Santana Formation (now considered Santana Group) is a well-known Konservat Lagerstätte in the Araripe Basin, northeastern Brazil, which is exceptionally rich in fossils (Maisey, 1991). The depositional sequence of the formation in the upper part of the Santana Group is mostly that of a fine-grained siliciclastic to carbonate-dominated series deposited during two transgressive–regressive cycles representing a marine post-rift incursion into the Araripe basin (Assine et al., 2014; Custódio et al., 2017; Fürsich et al., 2019). According to the low diversity benthic megafaunas preserved, the Romualdo Formation represents a high-stress environment (Fürsich et al., 2019). Considering only the vertebrate fossil record herein, several lineages such as crocodylomorphs, dinosaurs, and turtles are represented in the Santana Group, but it is arguably most famous for its exquisitely preserved and abundant bony fish and pterosaur faunas (Kellner et al., 2013; Martill, 2007; Sayão et al., 2020; Vila Nova et al., 2011; Wellnhofer, 1985).

## Material and methods

UFRPE 5061 is a forged concretion derived from the Romualdo Formation from Chapada do Araripe basin. As is often the case with outstanding lagerstätten-type localities, a market of dealing with fossils develops, with monetary, aesthetic or academic (vs. religious/shamanistic) reasons being the main drivers behind them (Ruffell et al., 2012). Local academics, national and international lawmakers, the international scientific community, institutes, and museums, as well as private and professional fossil collectors and dealers, bring forth their arguments, often conflicting among the involved parties, concerning the legal and illegal trade of fossils (Liston, 2014), even though the social and economic impacts of such trade have become more evident (Raja et al., 2021). Without going into detail, we recognise that this phenomenon is neither new nor localised (see for example Parham, 2005 on forged marine turtle remains from Morocco), thus, given the outstanding quality of many Santana fossils (Maisey, 1991), it is not surprising that a lucrative market for dealing in either genuine or forged fossils flourishes there as well (Martill, 1994). Despite the strict protection laws that have been emplaced in Brazil since 1942, which basically render all fossils—including the Santana fossils—as state property (see discussion in Cisneros et al., 2022), an abundance of specimens has been illegally exported to other countries worldwide over the last decades, mostly bony fish, as they are among the most common finds in the strata (Martill, 1994). Faking rarer specimens such as pterosaurs or dinosaurs might drastically increase their monetary value and often an array of methods and techniques are needed to tell genuine fossils and the frauds apart (Mateus et al., 2008; Veldmeijer, 2006). In general, the forged concretions lack stratigraphic data that would allow recognising spatial and temporal fluctuations of the vertebrate faunal composition (Vila Nova et al., 2011).

Via dubious trade ways, the specimen here described found its way to Europe and finally ended up in the collections of the Palaeontological Institute in Zurich, Switzerland, more than three decades ago (previously catalogued under PIMUZ A/III 619, now a cast of the original stored at PIMUZ). The concretion containing the turtle remains was not easily identified as having been forged at first, because of its weathered appearance and damaged external bone surface and thus it remained in the PIMUZ collection simply as a ‘Testudines indet.’ specimen.

**Fig. 1:**
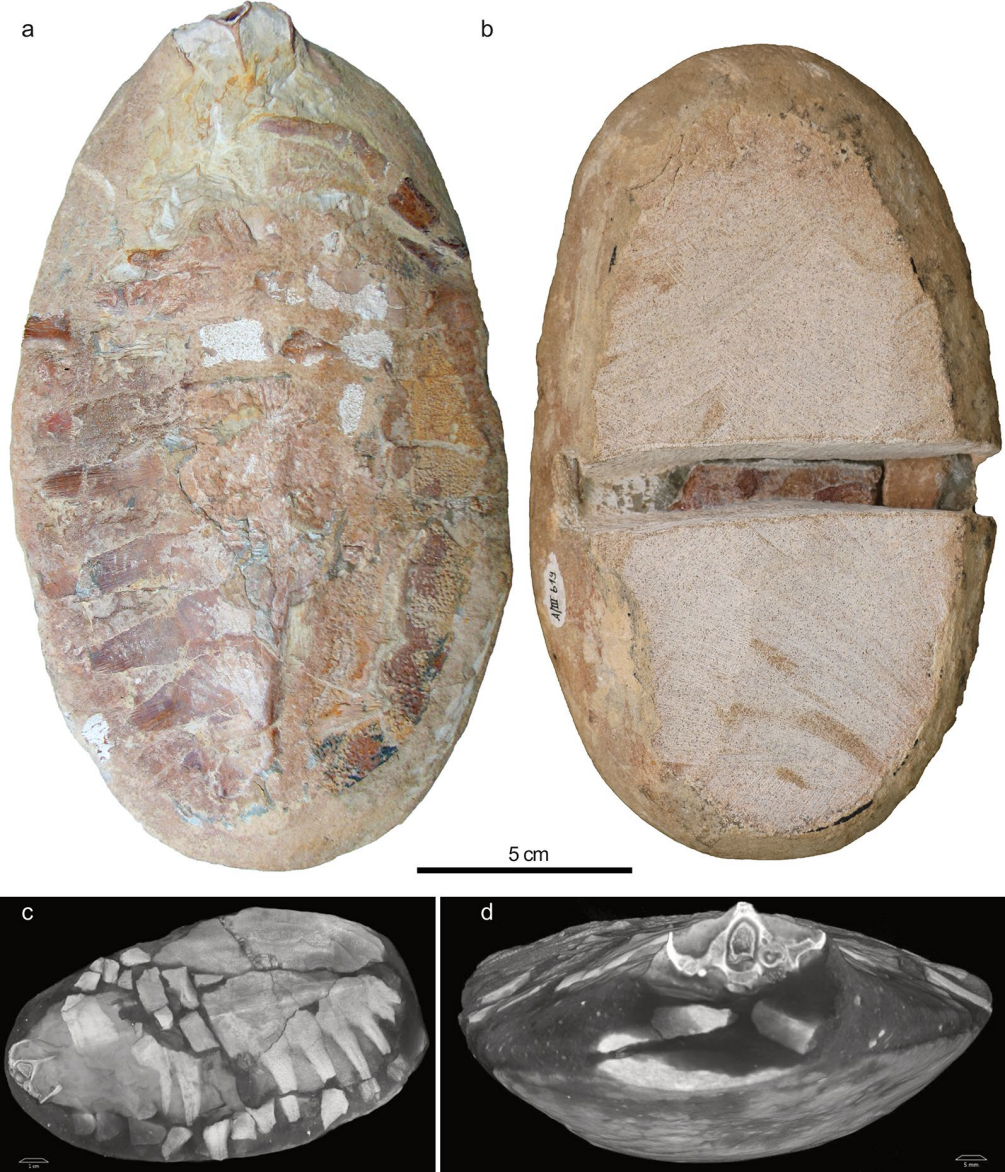
Forged concretion (UFRPE 5061) preserving parts of a new specimen of *Santanachelys* *gaffneyi* and *Araripemys* *barretoi* prior to preparation in dorsal view (**a**); during preparation of the ventral side (**b**); in angled dorsolateral (**c**) and anterior (**d**) visualisations of a first computed tomography scan. Note the differences in the colouration and structure of the sediment matrix at the base of the concretion

The reddish colour of the ventral side of the concretion turned out to be only a superficial layer of soft, burned-in dye covering a dense oval-shaped sedimentary matrix block. The specimen was first CT scanned in 2014 with a high-resolution peripheral quantitative CT (HR-pQCT, XtremeCT II, SCANCO Medical AG, Brüttisellen, Switzerland; see Additional video file) which yielded mixed results with low contrast and artefacts, mainly due to the dense sedimentary matrix. Especially the artefacts and low contrast prevented further segmentation of the bones. Scanning was performed at 68 kV tube voltage, 1470 μA tube current, 1.05 s integration time, and images were reconstructed using a 60.7μm isotropic voxel size. This scan, however, and initial preparation from the ventral side subsequently revealed that about the lower half of the specimen consisted of a very dense and coarse (artificial?) sediment block devoid of any bones (Fig. 1). This oval-shaped block was glued to the upper part of the specimen that contained the bones to give the impression of a concretion. Further preparation revealed that the whole concretion was thus assembled using synthetic car body filler of light grey colour and that at least two turtle specimens were included into the forgery. A similar case of forged turtle concretion was previously reported for specimen MN 6743-V of *Araripemys* (Oliveira, 2007) and it is, unfortunately, also a common practice among Araripe “peixeiros” (fishmongers; free translation from Portuguese).

The original sediment matrix containing most of the bones in UFRPE 5061 consists of a cream-coloured carbonate (forming the diagenetic concretion in which the fossils are preserved) that locally turns into coarser-grained carbonate matrix including scattered tiny bones and a partial articulated vertebral column of a tiny bony fish. Due to the continuity of this original matrix, the connection between the skull, neck and shell remains identified as *Santanachelys* could be confirmed. The remaining part of it was filled by shell bone pieces that were added haphazardly, often inside out, to give the impression of a more complete specimen. Due to the problematic nature of this Brazilian patrimonial specimen, it is now repatriated to the palaeontological collection of the Universidade Federal Rural de Pernambuco (UFRPE 5061), Brazil.

### Institutional abbreviations

DGM-DNPM, Divisão de Geologia e Mineralogia, Departamento Nacional de Produção Mineral, Rio de Janeiro, Brazil, currently, National Mining Agency (ANM). THUg, Teikyo Heisei University, Ichihara, Chiba, Japan. PIMUZ, Department of Palaeontology (formerly Palaeontological Institute and Museum), University of Zurich, Switzerland. UFRPE, Department of Biology, Universidade Federal Rural de Pernambuco, Recife, Brazil.

## Results

### Systematic palaeontology

*Pan-Cryptodira* Joyce et al., 2020 (Joyce et al., 2020a)

*Protostegidae* Cope, 1872 [(Joyce et al., 2021)]

*Santanachelys* *gaffneyi* Hirayama, 1998

#### Holotype

THUg1386, a nearly complete skeleton (Hirayama, 1998) [The specimen is currently housed in Fossil and Extant Turtle Collections in Waseda University, Tokyo, Japan].

#### Referred specimen

**Fig. 2:**
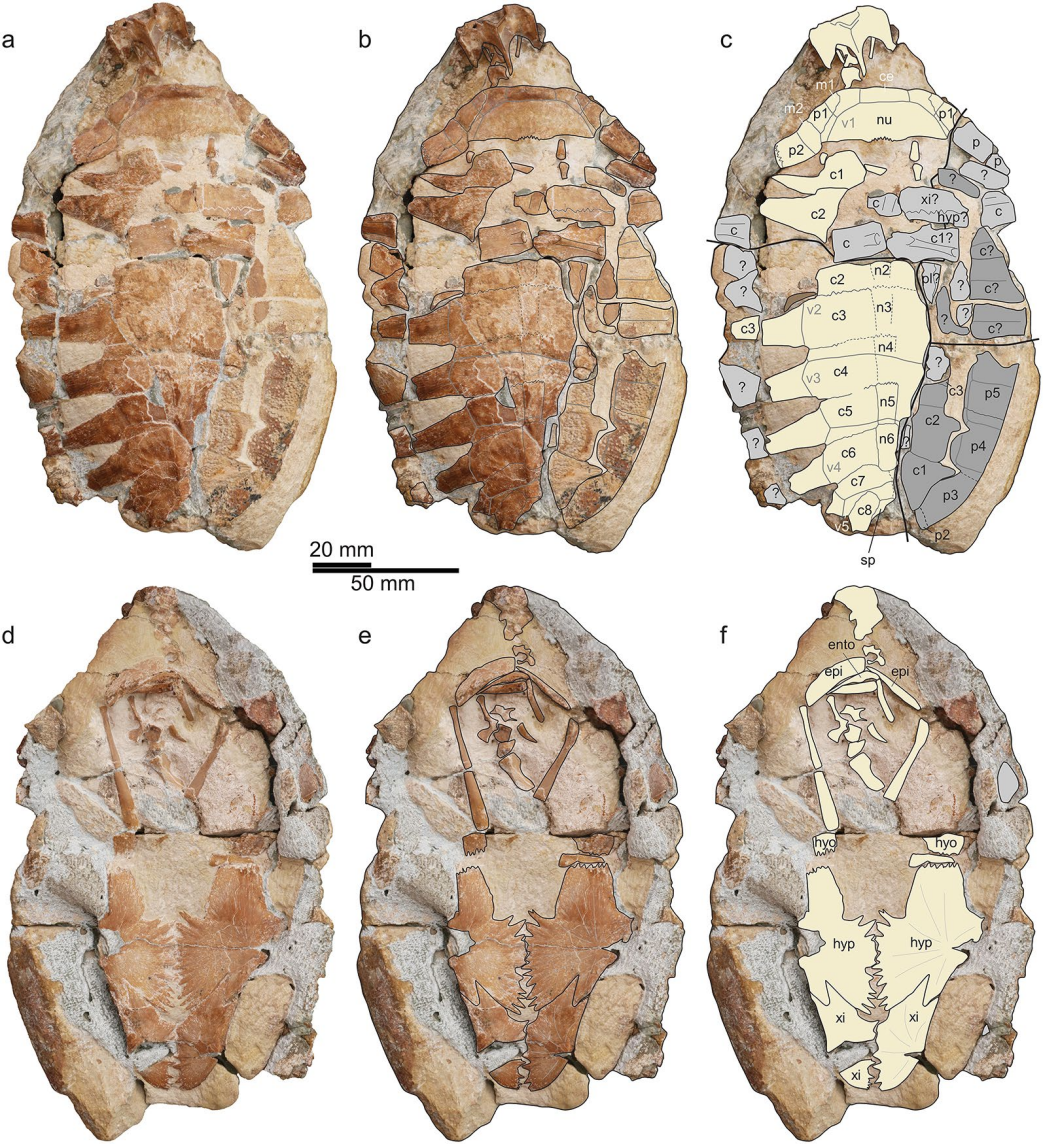
Forged concretion (UFRPE 5061) preserving parts of a new specimen of *Santanachelys* *gaffneyi* and *Araripemydidae* cf. *Araripemys* *barretoi* after preparation. **a-c** Specimen in dorsal view; **d-f** specimen in ventral view. **a, d** Complete fossil; **b, e** interpretative drawing of skeletal elements; **c, f** interpretative drawing with skeletal elements colour-coded according to identification as *Santanachelys* *gaffneyi* (light yellow), as belonging to cf. *Araripemys* *barretoi* (dark grey), and as unidentified shell elements (light grey). Of the latter, costal bones were identified because they were misleadingly included with the ventral view exposed (revealing parts of ribs). Abbreviations: c, costal bone; ce, cervical scute; epi, epiplastron; ento, entoplastron; hyo, hyoplastron; hyp, hypoplastron; m, marginal scute; p, peripheral bone; v, vertebral scute; xi, xiphiplastron

UFRPE 5061 (Fig. 2; Table 1), a forged concretion with a partial skeleton preserving the posterior part of the skull, neck, shoulder girdle, the nuchal, left and central parts of the carapace with few peripherals, and the plastron lacking most of the hyoplastra. These bones are identified as belonging to *Santanachelys* *gaffneyi* based on skull proportions, anteroposteriorly elongate oval shape of the carapace with large fontanelles between costals and peripherals, a faint radiating pattern and scute imprints on the dorsal surface of the costals, rectangular neurals, a plastron whose posterior part is identical in shape and suture pattern with the holotype specimen and framing a large central plastral fontanelle, elongate coracoids, and overall similar shell size compared to the holotype (although the latter alone is not considered a strong argument). UFRPE 5061 comes from Chapada do Araripe, Araripe basin, northeast of Brazil, but similar to the holotype specimen, no additional data about the finding situation and stratigraphic horizon are available for UFRPE 5061. Due to this lack of information, the age of the fossil is tentatively considered to be late Aptian (Arai and Assine, 2020; Fürsich et al., 2019; Melo et al., 2020).

**Table 1:** Measurements of UFRPE 5061 [in mm]

Maximum length of preserved bones in concretion	181.4
Maximum width of preserved bones in concretion	111.1
Maximum length of *Santanachelys* skull as preserved	19.4
Maximum width of *Santanachelys* skull	26.8
Maximum length of largest piece of *Santanachelys* shell	154.1
Maximum width of largest piece of *Santanachelys* shell	73.1
Anteroposterior length of *Santanachelys* nuchal	19.3
Width of *Santanachelys* nuchal (posterior margin)	44.30
Width of *Santanachelys* nuchal (anterior margin)	31.2
Length of *Santanachelys* right coracoid	44.1
*Santanachelys* left hypoplastron–xiphiplastron length	80.22
*Santanachelys* left xiphiplastron length	41.80
*Santanachelys* right epiplastron width	8.2
*Santanachelys* right epiplastron width	25.8
Maximum length of largest piece of Araripemydidae shell	106.1
Maximum width of largest piece of Araripemydidae shell	31.1

#### Description of UFRPE 5061

The majority of the preserved bones in the forged concretion could be identified as a partial skeleton of *Santanachelys* *gaffneyi*, including skull remains, appendicular and axial bones and shell parts. On the dorsal surface of the concretion, the posterior part of the skull is visible in dorsal view, followed posteriorly by a few cervicals that remain largely hidden within the matrix. These are followed by the carapace in dorsal view in which the nuchal, the right peripheral 1, the left peripherals 1 and 2, the lateral parts of left costal 1 partially overlapped by the lateral part of costal 2, the posteromedial part of costal 2, left costals 3–8, neurals 2–6, and the anterior left corner of a suprapygal bone could be identified. Much of the bone surface in the anterior part of the slab containing the posterior portion of costal 2 to costal 4 is strongly damaged so that sutures between bones are difficult to trace. There are fontanelles visible between the left costal 1 and peripheral 2, and between each of the left costals 3–8. On the ventral side of the concretion a small part of the skull, some cervicals mostly embedded in matrix, the two epiplastra, the partially preserved entoplastron, the posterior sutural areas of the hyoplastra, as well as mostly complete hypo- and xiphiplastra are preserved. The hyo- and hypoplastra frame a large central fontanelle of rectangular shape. The hypo- and xiphiplastra show a slight striation on the otherwise smooth bone surface that radiates out from the growth centre of each plate. We here focus on the diagnostic shell of the new specimen; other skeletal elements will be described and discussed elsewhere.

#### Skull, vertebrae, and shoulder girdle bones

The posterior portion of the skull is preserved and some bones such as the supraoccipital, opisthotics, and squamosals can be identified. Three cervical vertebrae are partially exposed, as are the very prominent elongated coracoids of the shoulder region. Parts of the scapula and additional dorsal centra are partly visible as well in ventral view, but because a full description of this specimen is currently in progress elsewhere, we refrain from adding anatomical details here.

#### Nuchal

The nuchal is completely preserved and contacts peripherals 1 and 2. This is in contrast to the holotype THUg1386, in which peripheral 1 extends slightly more caudally to the level of the posterior margin of the nuchal, and therefore hindering a contact with peripheral 2 (Hirayama, 1998; pers. obs. MR, TMS, and GSF). Posteriorly the margin of the nuchal is almost straight and medially the suture zone with the first neural is visible. Scute sulci reveal a rectangular wide cervical and adjacent marginal 1 and 2, as well as the vertebral 1.

#### Peripherals

The left peripherals 1 and 2 are completely preserved and there is a small remnant of peripheral 3 still sutured to peripheral 2. Peripheral 1 seems to be reduced in size and peripheral 2 more enlarged and massive compared to THUg1386. On the right side, only peripheral 1 is preserved. All peripherals show a slightly bowed indentation where the scute sulci meet the lateral shell margin.

#### Costals

The preserved lateral portion of costal 1 carries a long and tapering free rib end extending slightly anterolaterally. Most of its bone surface is abraded. The posterior margin of the element is largely overlapped by the anterior margin of the lateral portion of costal 2, with its free rib part extending anterolaterally. The separated posteromedial part of costal 2 is still sutured to neural 2 medially and costal 3 posteriorly. Costal 3 is mostly complete with a rectangular medial part, just lacking the tip of the tapering free rib end facing straight laterally (the distal part of the rib is broken off but fits well, unlike other fragments that have been added as supposed distal rib ends of costals 2, and 4–6). The bone surface is undamaged in the lateral part, preserving an anteroposteriorly extending sulcus of the vertebral 2. Costal 4 is of similar rectangular shape as costal 3 but its bone surface is less damaged, preserving a triple junction of sulci of vertebrals 2 and 3 close to the anterior margin of the free rib portion of the costal. The sulcus is traceable over the bone surface as a shallow groove and the free rib part is angled slightly posterolaterally. Costal 5 is narrower than costals 3 and 4, but still rectangular in shape with straight anterior, medial and posterior margins. The free rib is largely preserved and angled posterolaterally. The bone surface is almost undamaged except a small medial portion, preserving again a triple junction of vertebrals 3 and 4 situated more of the centre of the bone. Costal 6 is even narrower anteroposteriorly with straight anterior and medial margins, but a concave posterior one. The bone surface is undamaged, revealing the slightly bent lateral sulcus of vertebral 4. Costal 7 differs from the previous costals in almost having a crescent shape formed by a convex anterior margin, a straight to slightly undulating medial margin (where it does not articulate with a neural), a short straight posteromedial margin (i.e. the suture with the suprapygal), followed laterally by a strongly concave margin (i.e. the suture with costal 8). The small free rib end is only preserved proximally, extending strongly posterolaterally. The bone surface is undamaged revealing a triple junction between vertebrals 4 and 5. Costal 8 is much shorter and of rectangular shape, thus it is excluded from reaching the midline by the costal 7, but it has an anteromedial–posterolateral extending suture with the tiny preserved portion of the suprapygal. Its smooth bone surface lacks sulci.

#### Neurals

**Fig. 3:**
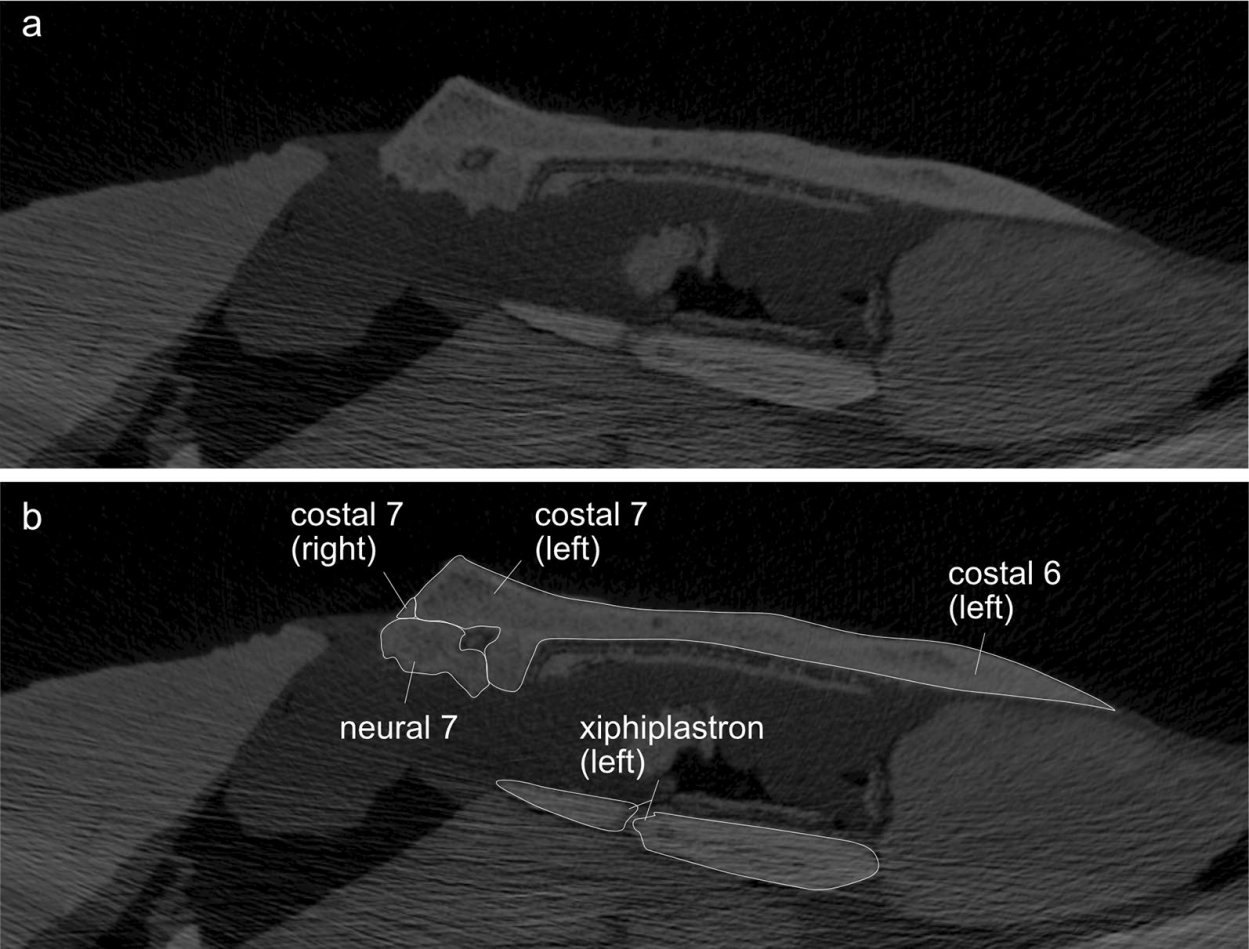
Close-up of image comprising the CT scan utilised in the present study (not to scale). **a** Cropped image; **b** image with interpretative drawing superimposed. The image shows a coronal (cross-sectional) cut through left costal 7 medially and costal 6 laterally. The suture between the costals extends not perpendicularly to the plane and is thus difficult to discern in the image. The rudimentary neural 7 is visible underneath the costals 7 that meet at the midline of the shell. The left xiphiplastron lies ventrally to costals 6 and 7

The neural series is continuous between neurals 2–6. Even though the lateral margins of the anterior neurals 2–4 cannot be traced completely with confidence, their outlines can at least still partially be reconstructed. Neural 1 is missing and the carapace appears to be lacking neurals posterior to number 6 in dorsal view, with costals 7 meeting in the midline in a straight to slightly undulating suture. While all neurals appear to be of elongate and narrow rectangular shape, only neurals 5 and 6 have an undamaged bone surface that allows precise shape identification. Neural 5 is hexagonal, with short sides anteriorly, whereas neural 6 is octagonal. A seventh neural is not exposed dorsally, but is rudimentarily developed beneath the costals 7 (Fig. 3).

#### Epiplastra

The epiplastra are slightly crescent-shaped bones with gently curved convex anterior and concave posterior margins. The elements have a straight midline margin and the lateral margin is slightly tapering, although the bones are eroded here.

#### Entoplastron

The entoplastron is T-shaped with a bowed convex anterior margin that articulates with the epiplastra, a medial posterior process of which only the proximal portion is preserved, and concave posterolateral margins. Given the separation of epiplastra and entoplastron from one another and the presence of smooth margins, the elements are interpreted as having been separate and not fused in life in UFRPE 5061.

#### Hyoplastra

Of the hyoplastra, only the posterior margins are preserved including the contact with the hypoplastra. Because of the missing parts of the hyoplastra, endoskeletal bones from the shoulder region (coracoids and scapulae) and at least three cervical vertebrae are exposed.

#### Hypoplastra

The hypoplastra have a complex shape that results from the anterior sutured margin with the hypoplastra, an anteromedial margin framing the posterior border of the central fontanelle, a strongly interdigitating medial suture, and a lateral margin which is strongly concave anterolaterally, more convex laterally (but broken in both bones) and straight to slightly concave posterolaterally. The posterior border forms a deep interdigitating socket for a xiphiplastral prong.

#### Xiphiplastra

The xiphiplastra meet one another in a strongly interdigitating midline suture. Their anterior margin is zigzag shaped as it forms prominent tapering prongs that articulate with the posterior margin of the hypoplastra. The lateral and posterior margins are convex and smoothly curved.

*Pleurodira* Cope, 1865 [(Joyce et al., 2020b)]

Araripemydidae Price, 1973

cf. *Araripemys* *barretoi* Price, 1973

#### Holotype

DGM-DNPM 756-R, a posterior portion of a shell and proximal hindlimb bones, found in the Romualdo Formation. The specimen is housed at the Agência Nacional de Mineração (National Mining Agency), previously called Departamento Nacional de Produção Mineral (DNPM), Rio de Janeiro, RJ, Brazil.

#### Referred specimen

UFRPE 5061, a forged concretion with a partial shell preserving the left costals 1–3 and peripherals 3–7. As for the rest of the concretion, no additional data are available. Identification is mainly based on the suture patterning of the costals and dorsal bone surface sculpturing consisting of fine pitting (Meylan, 1996, see description below).

#### Description of UFRPE 5061

The left anterior part of a carapace of an araripemydid, tentatively identified as belonging to cf. *Araripemys* *barretoi*, has been used to complete the forged concretion, with the anterior tip facing now posteriorly in relation to the *Santanachelys* *gaffneyi* remains. The distal portions of the left costals 1–3 and peripherals 2–5 are still in contact. Costal 1 and 2 also show the distal margins including the free rib ends as the costals and preserved peripherals are separated by fontanelles. The tip of the rib of costal 1 meets the suture between peripheral 3 and 4 as in *Araripemys* *barretoi* (see Meylan and Gaffney, 1991) but interestingly, the rib end of costal 2 is centrally situated, giving the costal a symmetrical straight shape rib being situated in the middle. Similar costal 2 shapes are also reported for *Laganemys* *tenerensis* (Sereno and ElShafie, 2013) and *Taquetochelys* *decorata* (Pérez-García, 2019), whereas in the well preserved AMNH 22550 of *Araripemys* *barretoi,* the rib lies acentrically in the anterior portion in the costal plate creating an asymmetrical costal shape in which the rib end converges slightly towards the suture with costal 1 (see also Meylan, 1996: fig. 3). In UFRPE 5061, the rib end of costal 2 further articulates with a pit centrally situated on peripheral 4; such an articulation was also shown in Meylan’s (1996) ventral view composite interpretative sketch of the carapace of AMNH 22550 (which differs from dorsal view skeletal reconstructions of the shell of *Araripemys* *barretoi* as shown in Meylan and Gaffney, 1991 and Sereno and ElShafie, 2013). Of costal 3, only a tiny part is visible, sutured to costal 2. The sutures between peripheral 2, 3 and 4 are only partially discernible, whereas there is a clear suture between peripheral 4 and 5. All bone surfaces appear strongly weathered and abraded, but a sculpturing pattern consisting of low knobs or protrusions is still distinctly visible on all bones. The presence of scute sulci is evidenced by low ridges, visible on peripheral 4 and 5, and on costal 2 just posterior to the free rib end. Two additional bone fragments (likely being parts of costals), separate from this larger slab, also carry a distinct bone surface sculpturing and are thus likely also referable to Araripemydidae; these bones are too fragmentary to be further identified. No bones referable to Araripemydidae are visible on the ventral side of the concretion.

### Indeterminate bone fragments

In addition, 18 small bone fragments on the dorsal side of the concretion appear to have been added haphazardly to fill holes between the larger shell parts mentioned above. Five of these have been identified as pertaining to costals being visible in ventral view (Fig. 2c), as they either preserve a proximal rib head or show the extent of the rib bulge within the costal. Two additional fragments likely also pertain to the plastron (one of which shows a strongly interdigitating suture between parts of a xiphiplastron and hypoplastron). Two fragments at the anterior right margin of the concretion were identified as peripherals due to their tapering cross-section, the anterolateral facing margin carrying sutural bony pegs, and the posteromedially facing margin being straight and tapering. The outer bone surface appears smooth (whether it is the dorsal or ventral bone surface remains unclear), unlike the dorsal bone surfaces of the peripherals of the *Santanachelys* or *Araripemys* peripherals. The remaining nine fragments could not be identified with confidence based on their shape or bone surface structure.

## Discussion and conclusions

UFRPE 5061 preserves a genuine partial skeleton including a posterior portion of the skull of the pan-cryptodiran protostegid marine turtle *Santanachelys* *gaffneyi* and represents the second known specimen of this key taxon. The missing part of the carapace was completed by carapace fragments of pleurodiran *Araripemydidae*, more common turtles of the Romualdo Formation, together with other fragments of indeterminate turtles. The estimated straight carapacial length of *Santanachelys* *gaffneyi* in UFRPE 5061 (ca. 170–180 mm; see Table 1) is slightly larger than that of the holotype specimen THUg1386 (145 mm; Hirayama, 1998). UFRPE 5061 further reveals that the new specimen has only six neurals dorsally exposed as a result of costals 7 and 8 meeting one another in the midline through a slightly undulating suture above neural 7. The remnant of neural 7, as is visible in the CT scan data (see Additional video file), thus appears to have been developmentally arrested earlier due to the costal overgrowth and thus lacks dorsal exposure. Concerning neural reduction in the turtle shell, Thomson and Georges (1996, p. 82) noted that “neural bones are probably structurally important for resisting downward pressure in high-domed species, but may be a disadvantage where lateral forces in flatter forms cause torsion among carapacial elements (Pritchard, 1988). Hence, strong swimmers that move by alternating thrusts of the rear limbs, and marine turtles that alternate strokes on land, tend to have reduced neural series with areas of median contiguity between opposing pleural bones (Pritchard, 1988)”. A negative allometry in the last neural bones in side-necked turtle *Bauruemys* *elegans* has been identified by Romano and Azevedo (2007), with neurals 5 and 6 being relatively smaller in larger adult specimens, and the sacral region of the carapace was identified as a more variable region in terms of shell bone pattern formation due to potential lack of connectivity with endoskeletal elements (Zangerl, 1969). Whether the developmental arresting of neural 7 in UFRPE 5061 is due to some specific functional aspects or similar behaviour as noted for the different aquatic species above, or whether it is a pathology/aberrant developmental feature or a simple intraspecific shape variation due to size (i.e., allometry) for the species cannot be elucidated at present. Finally, it is noteworthy that the vertebral 2–3 sulcus crosses neural 4 in UFRPE 5061, instead of crossing neural 3 as in the holotype THUg1386 (Hirayama, 1998), thus adding to the growing body of scute malformation and individual shield variation in turtles (e.g., Zimm, 2019). As only the second specimen of *Santanachelys* *gaffneyi* described, UFRPE 5061 thus provides first insights into morphological variation, as well as the potential to further study the skeletal anatomy of this taxon.

## Supplementary Information

**Additional file 1:** Flythrough video of the CT scan data produced at SCANCO Medical AG.

## Data Availability

The fossil specimen described here is available for study at the UFRPE and the cast of the specimen is stored at PIMUZ. All data generated or analysed during this study are included in this published article. The CT dataset is made available at Morphosource repository project ID 000501147 (https://www.morphosource.org/projects/000501147).

## References

[CR1] Anquetin J (2012). Reassessment of the phylogenetic interrelationships of basal turtles (Testudinata). Journal of Systematic Palaeontology.

[CR2] Anquetin J, Püntener C, Billon-Bruyat J-P (2015). *Portlandemys*
*gracilis* n. sp., a new coastal marine turtle from the Late Jurassic of Porrentruy (Switzerland) and a reconsideration of plesiochelyid cranial anatomy. PLoS ONE.

[CR3] Anquetin J, Püntener C, Joyce WG (2017). A review of the fossil record of turtles of the clade Thalassochelydia. Bulletin of the Peabody Museum of Natural History.

[CR4] Arai M, Assine ML (2020). Chronostratigraphic constraints and paleoenvironmental interpretation of the Romualdo Formation (Santana Group, Araripe Basin, Northeastern Brazil) based on palynology. Cretaceous Research.

[CR5] Assine ML, Perinotto JADJ, Custódio MA, Neumann VH, Varejão FG, Mescolotti PC (2014). Sequências deposicionais do Andar Alagoas da Bacia do Araripe, Nordeste do Brasil. Boletim De Geociências Da Petrobrás.

[CR6] Cadena EA, Parham JF (2015). Oldest known marine turtle? A new protostegid from the Lower Cretaceous of Colombia. PaleoBios.

[CR7] Carvalho ARA, Oliveira GR, Barreto AMF (2019). New occurrences of fossil Testudines of the Romualdo Formation, Aptian-Albian of the Araripe Basin, Pernambuco, Northeast Brazil. Journal of South American Earth Sciences.

[CR8] Cisneros JC, Raja NB, Ghilardi AM, Dunne EM, Pinheiro FL, Regalado Fernández OR, Sales MAF, Rodríguez-de la Rosa RA, Miranada-Martínez AY, González-Mora S, Bantim RAM, de Lima FJ, Pardo JD (2022). Digging deeper into colonial palaeontological practices in modern day Mexico and Brazil. Royal Society Open Science.

[CR9] Custódio MA, Quaglio F, Warren LV, GuimarãesSimões M, Fürsich FT, Perinotto JAJ, Assine ML (2017). The transgressive-regressive cycle of the Romualdo Formation (Araripe Basin): Sedimentary archive of the Early Cretaceous marine ingression in the interior of Northeast Brazil. Sedimentary Geology.

[CR10] Danilov IG, Parham JF (2006). A redescription of ‘*Plesiochelys*’ *tatsuensis* from the Late Jurassic of China, with comments on the antiquity of the crown clade Cryptodira. Journal of Vertebrate Paleontology.

[CR11] Evers SW, Barrett PM, Benson RBJ (2019). Anatomy of *Rhinochelys*
*pulchriceps* (Protostegidae) and marine adaptation during the early evolution of chelonioids. PeerJ.

[CR12] Evers SW, Benson RBJ (2019). A new phylogenetic hypothesis of turtles with implications for the timing and number of evolutionary transitions to marine lifestyles in the group. Palaeontology.

[CR13] Fürsich FT, Custódio MA, Matos SA, Hethke M, Quaglio F, Warren LV, Assine ML, Simões MG (2019). Analysis of a Cretaceous (late Aptian) high-stress ecosystem: The Romualdo Formation of the Araripe Basin, northeastern Brazil. Cretaceous Research.

[CR14] Gaffney ES (2001). *Cearachelys*, a new side-necked turtle (Pelomedusoides: Bothremydidae) from the Early Cretaceous of Brazil. American Museum Novitates.

[CR15] Gaffney ES, Tong H, Meylan PA (2006). Evolution of the side-necked turtles: The families Bothremydidae, Euraxemydidae, and Araripemydidae. Bulletin of the American Museum of Natural History.

[CR16] Gaffney, E. S., Meylan, P. A., Wood, R. C., Simons, E., & Almeida Campos, D. de. ( 2011). Evolution of the side-necked turtles: the family Podocnemididae. *American Museum of Natural History, Bulletin*, *350,* 1–237. 10.1206/350.1

[CR17] Gentry AD, Ebersole JA, Kiernan CR (2019). *Asmodochelys*
*parhami*, a new fossil marine turtle from the Campanian Demopolis Chalk and the stratigraphic congruence of competing marine turtle phylogenies. Royal Society Open Science.

[CR18] Hirayama R (1998). Oldest known sea turtle. Nature.

[CR19] Joyce WG (2007). Phylogenetic relationships of Mesozoic turtles. Bulletin of the Peabody Museum of Natural History.

[CR20] Joyce WG, Anquetin J, Cadena E-A, Claude J, Danilov IG, Evers SW, Ferreira GS, Gentry AD, Georgalis GL, Lyson TR, Pérez-García A, Rabi M, Sterli J, Vitek NS, Parham JF (2021). A nomenclature for fossil and living turtles using phylogenetically defined clade names. Swiss Journal of Palaeontology.

[CR21] Joyce WG, Parham JF, Anquetin J, Claude J, Danilov I, Iverson JB, Kear BP, Lyson TR, Rabi M, Sterli J, de Queiroz K, Cantino PD, Gauthier JA (2020). Pan-Cryptodira. Phylonyms—A Companion to the PhyloCode.

[CR22] Joyce WG, Parham JF, Anquetin J, Claude J, Danilov I, Iverson JB, Kear BP, Lyson TR, Rabi M, Sterli J, de Queiroz K, Cantino PD, Gauthier JA (2020). Pleurodira. Phylonyms—A Companion to the PhyloCode.

[CR23] Kear BP, Lee MSY (2006). A primitive protostegid from Australia and early sea turtle evolution. Biology Letters.

[CR24] Kellner AWA, Campos DA, Sayão JM, Saraiva AÁF, Rodrigues T, Oliveira GR, Cruz LA, Costa FR, Silva HP, Ferreira JS (2013). The largest flying reptile from Gondwana: A new specimen of *Tropeognathus* cf. *T.*
*mesembrinus* Wellnhofer, 1987 (Pterodactyloidea, Anhangueridae) and other large pterosaurs from the Romualdo Formation, Lower Cretaceous, Brazil. Anais Da Academia Brasileira De Ciências.

[CR25] Lapparent F, de Broin F (2000). The oldest pre-podocnemidid turtle (Chelonii, Pleurodira), from the early Cretaceous, Ceará state, Brasil, and its environment. Treballs Del Museu De Geologia De Barcelona.

[CR26] Limaverde S, Vargas Pêgas R, Damasceno R, Villa C, Oliveira GR, Bonde N, Leal MEC (2020). Interpreting character variation in turtles: *Araripemys*
*barretoi* (Pleurodira: Pelomedusoides) from the Araripe Basin, Early Cretaceous of Northeastern Brazil. PeerJ.

[CR27] Liston J (2014). Fossil protection legislation: Chinese issues, global problems. Biological Journal of the Linnean Society.

[CR28] Maisey, J. G. (Ed.) (1991). *Santana**fossils:**An**Illustrated**Atlas*. Neptune City: T.F.H. Publications.

[CR29] Martill DM (1994). Fake fossils from Brazil. Geology Today.

[CR30] Martill DM (2007). The age of the Cretaceous Santana Formation fossil Konservat Lagerstätte of north-east Brazil: A historical review and an appraisal of the biochronostratigraphic utility of its palaeobiota. Cretaceous Research.

[CR31] Mateus O, Overbeeke M, Rita F (2008). Dinosaur frauds, hoaxes and "Frankensteins": How to distinguish fake and genuine vertebrate fossils. Journal of Paleontological Techniques.

[CR32] Melo RM, Guzmán J, Almeida-Lima D, Piovesan EK, Neumann VHML, Sousa AJ (2020). New marine data and age accuracy of the Romualdo Formation, Araripe Basin, Brazil. Scientific Reports.

[CR33] Meylan PA (1996). Skeletal morphology and relationships of the Early Cretaceous side-necked turtle, *Araripemys*
*barretoi* (Testudines: Pelomedusoides: Araripemydidae), from the Santana Formation of Brazil. Journal of Vertebrate Paleontology.

[CR34] Meylan P, Gaffney ES, Maisey JG (1991). *Araripemys* PRICE, 1973. Santana fossils: An illustrated atlas.

[CR35] Oliveira GR (2007). Aspectos Tafonômicos de Testudines da Formação Santana (Cretáceo Inferior), Bacia do Araripe, Nordeste do Brasil. Anuário Do Instituto De Geociências UFRJ, Brazil.

[CR36] Oliveira GR, Kellner AWA (2005). Note on a plastron (Testudines, Pleurodira) from the Lower Cretaceous Crato Member, Santana Formation, Brazil. Arquivos Do Museu Nacional, Rio De Janeiro.

[CR37] Oliveira GR, Kellner AWA (2007). A new side-necked turtle (Pleurodira, Pelomedusoides) from the Santana Formation (Early Cretaceous), Araripe Basin, Northeastern Brazil. Zootaxa.

[CR38] Oliveira GR, Kellner AWA (2017). Rare hatchling specimens of *Araripemys* Price, 1973 (Testudines, Pelomedusoides, Araripemydidae) from the Crato Formation, Araripe Basin. Journal of South American Earth Sciences.

[CR39] Oliveira GR, Romano PSR (2007). Histórico dos achados de tartarugas fósseis do Brasil. Arquivos Do Museu Nacional, Rio De Janeiro.

[CR40] Oliveira GR, Saraiva AAF, De Paula Silva H, Gomes de Antrade JAF, Kellner AWA (2011). First turtle from the Ipubi Formation (Early Cretaceous), Santana Group, Araripe Basin, Brazil. Revista Brasileira De Paleontologia.

[CR41] Parham JF (2005). A reassessment of the referral of the sea turtle skulls to the genus *Osteopygis* (Late Cretaceous, New Jersey, USA). Journal of Vertebrate Paleontology.

[CR42] Pérez-García, A. (2019). Identification of the Lower Cretaceous pleurodiran turtle *Taquetochelys decorata* as the only African araripemydid species. *Comptes Rendus Palevol,**18, *24–32. 10.1016/j.crpv.2018.04.004

[CR43] Price LI (1973). Quelônio Amphichelydia no Cretáceo Inferior do Nordeste do Brasil. Revista Brasileira De Geociências.

[CR44] Pritchard PCH (1988). A survey of neural bone variation among recent chelonian species, with functional interpretations. Acta Zoologica Cracoviensia.

[CR45] Rabi M, Kear BP (2016). Transitional fossils shed light on the basal divergence of advanced marine turtles. Journal of Vertebrate Paleontology, Program and Abstracts Book.

[CR46] Raja NB, Dunne EM, Matiwane A, Khan TM, Nätscher PS, Ghilardi AM, Chattopadhyay D (2021). Colonial history and global economics distort our understanding of deep-time biodiversity. Nature Ecology and Evolution.

[CR47] Raselli Irena (2018). Comparative cranial morphology of the Late Cretaceous protostegid sea turtle Desmatochelys lowii. PeerJ.

[CR48] Romano, P. S. R., Oliveira, G. R., Azevedo, S. A. K., Kellner, A. W. A., & de Almeida Campos, D. (2013). New Information about Pelomedusoides (Testudines: Pleurodira) from the Cretaceous of Brazil. In: Brinkman, D. B., Holroyd, P. A., & Gardner, J. D. (Eds.), *Morphology**and**Evolution**of**Turtles.**Proceedings**of**the**Gaffney**Turtle**Symposium**(2009)**in**Honor**of**Eugene**S**Gaffney.**Vertebrate**Paleobiology**and**Paleoanthropology**Series* (pp. 261–275). Dordrecht: Springer.

[CR49] Romano PS, Azevedo SAK (2007). Morphometric analysis of the Upper Cretaceous Brazilian side-necked turtle *Bauruemys*
*elegans* (Suárez, 1969) (Pleurodira, Podocnemididae). Arquivos Do Museu Nacional.

[CR50] Ruffell A, Majury N, Brooks WE (2012). Geological fakes and frauds. Earth-Science Reviews.

[CR51] Sayão JM, Feitosa Saraiva AÁ, Brum AS, Bantim RAM, Pedroso de Andrade RCL, Xin C, Jorge de Lima F, De Paula Silva H, Kellner AWA (2020). The first theropod dinosaur (Coelurosauria, Theropoda) from the base of the Romualdo Formation (Albian), Araripe Basin, Northeast Brazil. Scientific Reports.

[CR52] Sena MVA, Bantim RAM, Saraiva AAF, Sayão JM, Oliveira GR (2021). Osteohistology and microanatomy of a new specimen of *Cearachelys*
*placidoi* (Testudines: Pleurodira) a side-necked turtle from the Lower Cretaceous of Brazil. The Anatomical Record.

[CR53] Sereno, P. C., ElShafie, S. J. (2013. A new long-necked turtle *Laganemys tenerensis *(Pleurodira: Araripemydidae) from the Elrhaz Formation (Aptian–Albian) of Niger. In: Brinkman, D. B., Holroyd, P. A., & Gardner, J. D. (Eds.), Morphology and Evolution of Turtles. Proceedings of the Gaffney Turtle Symposium (2009) in Honor of Eugene S Gaffney. Vertebrate Paleobiology and Paleoanthropology Series (pp. 215–250). Dordrecht: Springer.

[CR54] Sterli J (2010). Phylogenetic relationships among extinct and extant turtles: The position of Pleurodira and the effects of the fossils on rooting crown-group turtles. Contributions to Zoology.

[CR55] Sterli J, de La Fuente M (2011). A new turtle from the La Colonia Formation (Campanian-Maastrichtian), Patagonia, Argentina, with remarks on the evolution of the vertebral column in turtles. Palaeontology.

[CR56] Thomson S, Georges A (1996). Neural bones in Australian chelid turtles. Chelonian Conservation and Biology.

[CR57] Veldmeijer, A. J. (2006). Toothed pterosaurs from the Santana Formation (Cretaceous; Aptian–Albian) of northeastern Brazil. A reappraisal on the basis of newly described material. *PhD**Thesis,**Universiteit**Utrecht,**The**Netherlands.* pp. 269.

[CR58] Vila Nova BC, Saraiva AAF, Moreira JKR, Sayão JM (2011). Controlled excavations in the Romualdo Formation Lagerstätte (Araripe Basin, Brazil) and pterosaur diversity: Remarks based on new findings. Palaios.

[CR59] Wellnhofer P (1985). Neue Pterosaurier aus der Santana-Formation (Apt) der Chapada do Araripe, Brasilien. Palaeontographica Abt. A.

[CR60] Zangerl R, Gans C, Bellairs ADA, Parsons TS (1969). The turtle shell. Biology of the Reptilia. Morphology A.

[CR61] Zimm, R. (2019). On the development of the turtle scute pattern and the origins of its variation. *PhD**thesis,**Institute**of**Biotechnology,**Department**of**Biosciences,**University**of**Helsinki,**Finland*. pp. 89.

